# Unfolding anti-tumor immunity: ER stress responses sculpt tolerogenic myeloid cells in cancer

**DOI:** 10.1186/s40425-016-0203-4

**Published:** 2017-01-17

**Authors:** Juan R. Cubillos-Ruiz, Eslam Mohamed, Paulo C. Rodriguez

**Affiliations:** 1 Weill Cornell Medicine, Department of Obstetrics & Gynecology, Sandra and Edward Meyer Cancer Center, 1300 York Ave, E-907, New York, NY 10065 USA; 2 Georgia Cancer Center, Augusta University, 1410 Laney Walker Blvd, Room CN-4125A, Augusta, GA 30912 USA; 3 Department of Medicine, Georgia Cancer Center, Augusta University, 1410 Laney Walker Blvd, Room CN-4114, Augusta, GA 30912 USA

**Keywords:** ER stress, Unfolded Protein Responses, IRE1, XBP1, PERK, CHOP, Myeloid cells, Immunotherapy, Tumor immunology

## Abstract

Established tumors build a stressful and hostile microenvironment that blocks the development of protective innate and adaptive immune responses. Different subsets of immunoregulatory myeloid populations, including dendritic cells, myeloid-derived suppressor cells (MDSCs) and macrophages, accumulate in the stressed tumor milieu and represent a major impediment to the success of various forms of cancer immunotherapy. Specific conditions and factors within tumor masses, including hypoxia, nutrient starvation, low pH, and increased levels of free radicals, provoke a state of “endoplasmic reticulum (ER) stress” in both malignant cells and infiltrating myeloid cells. In order to cope with ER stress, cancer cells and tumor-associated myeloid cells activate an integrated signaling pathway known as the Unfolded Protein Response (UPR), which promotes cell survival and adaptation under adverse environmental conditions. However, the UPR can also induce cell death under unresolved levels of ER stress. Three branches of the UPR have been described, including the activation of the inositol-requiring enzyme 1 (IRE1), the pancreatic ER kinase (PKR)-like ER kinase (PERK), and the activating transcription factor 6 (ATF6). In this minireview, we briefly discuss the role of ER stress and specific UPR mediators in tumor development, growth and metastasis. In addition, we describe how sustained ER stress responses operate as key mediators of chronic inflammation and immune suppression within tumors. Finally, we discuss multiple pharmacological approaches that overcome the immunosuppressive effect of the UPR in tumors, and that could potentially enhance the efficacy of cancer immunotherapies by reprogramming the function of tumor-infiltrating myeloid cells.

## Background

The Endoplasmic Reticulum (ER) plays a fundamental role in the homeostatic synthesis, folding and glycosylation of nascent transmembrane and secretory proteins [1]. In addition, the ER acts as the primary organelle for calcium storage and biosynthesis of lipids and sterols in eukaryotic cells [2]. The physiological activity of the ER is tightly controlled by intrinsic processes such as cell differentiation, proliferation status and activation signals, as well as by exogenous factors in the microenvironment [3]. For instance, hostile conditions in the tumor milieu such as hypoxia, nutrient starvation, low pH, and free radicals can rapidly disrupt the protein folding capacity of the ER, thereby triggering a state of cellular “ER stress” [4]. The accumulation of misfolded proteins in the ER activates the Unfolded Protein Response (UPR), which is an integrated signaling pathway that attempts to restore the homeostasis of this organelle. The UPR drives multiple adaptive and survival processes, including the attenuation of *de novo* protein synthesis, the regulation of the ER membrane, the degradation of misfolded proteins, and the selective induction of mediators and chaperones that promote the correct folding of proteins [5]. However, when ER stress is severe and prolonged, the same UPR mediators that regulate survival can trigger the induction of cellular death [6]. Overactivation of UPR mediators has been implicated in several pathological processes, including cancer, diabetes, and cardiovascular and neurodegenerative diseases [4]. In addition, recent studies have demonstrated the importance of the UPR in the overall modulation of chronic inflammation in cancer [7–10]. In this review, we discuss how ER stress and aberrant activation of the UPR alter the function of malignant cells and cancer-associated myeloid cells, and how this process controls anti-tumor immunity. We also discuss various pharmacological approaches to overcome the immunosuppressive effect of ER stress in tumors and the potential of these strategies as new cancer immunotherapies.

## Review

### ER stress sensors and the UPR

The UPR plays a crucial role in mediating cellular adaptation to ER stress. Three major ER-localized transmembrane proteins trigger this adaptive pathway: the inositol-requiring enzyme 1 (IRE1), the pancreatic ER kinase (PKR)-like ER kinase (PERK), and the activating transcription factor 6 (ATF6) [4]. In the absence of ER stress, these three sensors are bound and maintained in an inactive form by the HSP70-type chaperone BiP/GRP78 [11–13]. Because BiP exhibits a higher affinity for misfolded proteins, the induction of ER stress causes the dissociation of BiP from the sensors, leading to their activation and subsequent initiation of the UPR. The mechanisms by which the major mediators of the UPR regulate cellular responses under ER stress are as follow:

### IRE1

The Type I ER transmembrane protein IRE1 is a dual enzyme with serine/threonine-protein kinase and endoribonuclease activity that exists in two conserved isoforms: IRE1α and IRE1β [14, 15]. IRE1α is ubiquitously expressed, whereas IRE1β expression is limited to the gut [14, 16]. At steady state, the chaperone BiP maintains IRE1α in its monomeric form, thereby impeding its activation. During ER stress, the accumulation of misfolded proteins titrate BiP away from IRE1α, allowing IRE1α dimerization, autophosphorylation, and a conformational shift that licenses its C-terminal endoribonuclease domain to excise 26 nucleotides from the X-box binding protein 1 (*Xbp1*) mRNA in the cytosol [17–19]. The spliced transcript is subsequently re-ligated by the tRNA ligase RtcB [20], resulting in a critical reading frame shift that allows the generation of the functionally mature XBP1. This transcription factor effectively alleviates ER stress by inducing the expression of chaperones, redox-dependent foldases, and glycosyltransferases. Beyond its canonical functions in the UPR, XBP1 can also modulate ER stress-independent, context-specific processes such as response to hypoxia [21], lipid metabolism [22], estrogen receptor activity [23] and the transcriptional induction of pro-inflammatory cytokines [24], among many others.

Although most of the IRE1α signaling events are associated with the induction of pro-survival pathways, IRE1α can also trigger apoptosis under severe or lethal ER stress. As such, IRE1α can degrade non-*Xbp1* mRNA targets through regulated IRE1α-dependent decay (RIDD), a phenomenon that has been previously associated with the induction of apoptosis [25]. Moreover, active IRE1α complexes with the adaptor protein TNF-receptor-associated factor 2 (TRAF2), which recruits the apoptosis-signal-regulating kinase (ASK1), leading to cell death or autophagy [26–28]. Additionally, IRE1α-linked apoptosis has been reported to be mediated through the activation of the c-Jun N-terminal kinase (JNK) and a subsequent inhibition of BCL2 family members [29]. Furthermore, activation of XBP1 through IRE1α induces the expression of the HSP40 family member P58IPK, which binds and inhibits PERK, overcoming the PERK-mediated translational block [30]. Although this event can represent the termination of the UPR under transient ER stress, it may also trigger apoptosis under severe conditions of stress through the translation of pro-apoptotic mediators [31, 32]. Thus, IRE1α can play a dual role in the cellular responses against ER stress by promoting both survival and cell death.

### PERK

Under homeostatic conditions, the type I ER transmembrane protein PERK (or eIF2aK3) is maintained in an inactive form also through complexing with BiP [33]. After the induction of ER stress and release of BiP, PERK activates through oligomerization and autophosphorylation, leading to the phosphorylation of various PERK substrates, including the eukaryotic translation initiation factor 2 alpha (eIF2α), the NF-E2-related factor 2 (Nrf2), the forkhead box O proteins (FOXO), and the second messenger diacyglycerol (DAG) [34]. The increased susceptibility of PERK null primary cells and tumor cells to ER stress-induced cell death suggests the major role of PERK in pro-survival mechanisms [35, 36]. The best-characterized PERK-linked effect is the phosphorylation of eIF2α, which serves as a common regulator of the integrated stress responses in cells. In addition to PERK, three different kinases, the double-stranded RNA-dependent protein kinase (PKR), the hemin-regulated inhibitor (HRI), and the nutrient starvation activated kinase GCN2, phosphorylate eIF2α in response to specific forms of stress [37]. Phospho-eIF2α inhibits nucleotide exchange on the eIF2 complex, attenuating translation of most mRNAs, thereby alleviating additional sources of ER stress [37]. In addition, it increases the Cap-independent expression of a limited number of proteins that eventually control the cell fate during stress, including the activating transcription factor 4 (ATF4). Thus, phosphorylation of eIF2α by PERK serves as a major mechanism to decrease protein synthesis and thereby counter the accumulation of misfolded proteins in the stressed ER. In addition, active PERK phosphorylates Nrf2, which then translocates to the nucleus and induces the expression of multiple cellular redox transcripts that alleviate the effects of stress-induced reactive oxygen species (ROS) [38]. Also, activation of FOXO proteins by PERK negatively regulates AKT activity and therefore converts stressed cells from anabolic metabolic programs into those leading to nutrient catabolism [39]. Thus, the activation of PERK plays a fundamental role in the metabolic adaptation of cells to ER stress.

Phosphorylation of eIF2α induces the activation of ATF4 that directly regulates the survival of the stressed cells through the induction of autophagy. Interestingly, ATF4 induction after uncontrolled or chronic ER stress regulates the expression of the pro-apoptotic protein CAAT/enhancer binding protein (C/EBP) homologous protein (CHOP/Ddit3), which plays a key role in the induction of cell death by stress [40]. The mechanism by which PERK activity plays a dual role in the survival of stressed cells has been recently demonstrated. The induction of ATF4 after PERK activation results in the transient expression of the microRNA miR-211, which temporarily blocks the transcription of pro-apoptotic CHOP. However, after the expiration of miR-211, CHOP transcription proceeds and the cells undergo apoptosis [41]. Therefore, similar to the role played by IRE1α, the activation of PERK can mediate pro-survival or pro-apoptotic effects.

### ATF6

ATF6 is an ER-resident type II transmembrane protein that exists as 2 homologs (ATF6α and ATF6β) and serves as a precursor for a cytoplasmic N-terminal bZIP transcription factor [42]. Upon dissociation from BiP, ATF6α translocates to the Golgi apparatus via coat protein COPII–covered vesicles where it results cleaved by site 1 and site 2 proteases, enabling its transcription factor potential [13]. ATF6α target genes regulate the folding and glycosylation of *de novo* proteins, thereby regulating the survival of stressed cells [43]. In addition, several common targets of ATF6α are also regulated by XBP1, suggesting potential overlapping effects of IRE1α and ATF6α. Although the role of ATF6α and ATF6β upon ER stress remains less critical than that induced by IRE1α and PERK, the knockdown of *Atf6* results in lower survival rates after specific chemically-induced ER stress, indicating that ATF6α is indeed protective in the responses induced by pharmacological ER stress [43].

### Role of the UPR in malignant cells

The key interaction between the UPR and tumorigenesis has been comprehensively discussed in previous reviews [1, 4, 5, 34]. Malignant cells thrive under ER stress-inducing conditions such as hypoxia, nutrient deprivation, and low pH. In addition, cancer cells generate reactive metabolic byproducts that avidly modify ER-resident proteins and chaperones. Notably, the induction of various UPR-related factors has been commonly reported in patients with various cancer types and their overexpression usually correlates with poor prognosis and resistance to therapy [21, 44–46]. Interestingly, treatment of tumor-bearing mice with the ER stress inducer thapsigargin increased tumor growth, whereas global UPR inhibition using chemical chaperones, such as 4-Phenylbutyric acid (4-PBA) or tauroursodeoxycholic acid (TUDCA), delayed tumor progression and metastasis [9, 47].

Seminal studies have determined the cancer cell-intrinsic protumoral role of the IRE1α- XBP1 and the PERK-eIF2α pathways in vivo. Implantation of malignant cells or transformed fibroblasts lacking IRE1α/XBP1 or PERK/eIF2α in mice resulted in reduced tumor growth, which was attributed to low angiogenesis and increased sensitivity of the cancer cells to ER stress inducers, including hypoxia and high levels of ROS [35]. Accordingly, targeting IRE1α or PERK signaling in vivo with specific small-molecule inhibitors has shown significant therapeutic effects in various preclinical models of disease [48–52]. More recently, XBP1 was demonstrated to foster triple negative breast cancer progression by cooperating with HIF1α to support tumor-initiating cell function and metastatic capacity under hypoxia [21]. XBP1 contributes to the pathogenesis of multiple myeloma [53], and has been implicated in cancer cell de-differentiation, susceptibility to oncovirus infection and the epithelial-to-mesenchymal transition [54]. Andrew Hu and colleagues have elegantly demonstrated constitutive IRE1α-XBP1 activation in chronic lymphocytic leukemia cells, which promoted their pathogenesis in vivo [48]. In addition, inhibiting IRE1α function by overexpressing a dominant negative IRE1α variant significantly increased overall host survival by decreasing tumor growth rate and angiogenesis in a model of glioma [55]. Recent studies have also indicated that IRE1α-XBP1 signaling supports the aggressiveness of pancreatic cancer cells in xenograft models [56].

Similar to the effect induced by IRE1α-XBP1 signaling, the activation of PERK-eIF2α has also been implicated in the development of several malignancies, including breast, lung, and liver carcinoma [36, 47]. In those models, deletion of *Perk* rendered malignant cells highly susceptible to the cell death induced after exposure to hypoxia, DNA damage, low levels of nutrients, and high levels of reactive oxygen species [57]. Furthermore, the absence of PERK-eIF2α signaling impaired the ability of breast cancer cells to migrate and invade, thereby decreasing their ability to metastasize in vivo [49, 58, 59]. Therefore, the inhibition of PERK resulted in cancer cell apoptosis and significant anti-tumor effects [43]. As such, silencing of *Perk* increased the therapeutic efficacy of treatments based on the depletion of amino acids in T cell leukemia [60], and sensitized chronic myeloid leukemia (CML) cells to the apoptosis induced by the BCR/ABL inhibitor, imatinib mesylate [61]. Thus, the intrinsic effects of a controlled UPR in cancer cells appear to favor tumor growth and metastasis through the promotion of malignant cell survival, angiogenesis and chemoresistance, thus justifying the use of specific UPR inhibitors for the treatment cancer.

Although activation of the UPR has been primarily associated with cancer cell survival and tumor progression, some studies suggest that molecular factors in this pathway could also suppress tumor development in certain contexts. For instance, increased oncogenic transformation has been evidenced in fibroblasts after inhibiting the PERK target eIF2α [62], and increased proliferation and mammary tumor formation has been reported upon expression of a dominant-negative form of PERK in mammary epithelial cells [63]. Furthermore, in the context of acute myeloid leukemia, increased expression of ER stress response markers correlates with better prognosis in patients with this disease [64]. Taken together, these studies indicate that the effects of the UPR in cancer cells is context-dependent and that variables such as the stage of cancer progression and the cellular source of malignancy are critical determinants of whether this pathway plays either a pro-tumorigenic or anti-tumoral role.

### ER-stressed cancer cells efficiently manipulate myeloid functions

**Fig. 1:**
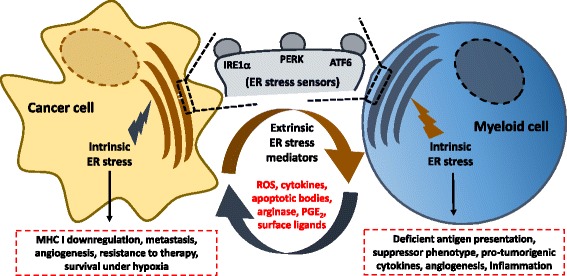
Hostile conditions in the tumor microenvironment such as hypoxia, nutrient deprivation and ROS can provoke ER stress and trigger the UPR in various tumor-resident cell types. Intrinsic ER stress responses in cancer cells ensure their survival under hypoxic conditions, increase expression of pro-angiogenic factors, promote metastasis and inhibit the presentation of their own antigens. Myeloid-intrinsic ER stress responses mediate reprogramming towards immunosuppressive and tolerogenic phenotypes. Induction of ER stress in myeloid cells may occur via transmissible factors released by ER-stressed cancer cells in the same milieu. Intracellular generation and accumulation of lipid peroxidation byproducts can further elicit intrinsic ER stress responses in myeloid cells. ER stress sensors therefore emerge as attractive targets for developing new immunotherapeutic approaches that may synergize with standard cancer treatments

Although the effect of the UPR in the survival/death of malignant cells has been extensively studied during the last decade, its role in the modulation of anti-tumor immunity has remained minimally characterized. Superior tolerogenic activity is observed in tumor-infiltrating myeloid cells compared with those located outside the tumors, suggesting a role for the tumor-stressed microenvironment in the control of myeloid cell function [65, 66]. Initial in vitro studies reported paracrine effects of tumor cells undergoing ER stress on dendritic cells (DCs), macrophages, and myeloid-derived suppressor cells (MDSCs). Pharmacological induction of UPR in cancer cells triggered “transmissible” ER stress in myeloid cells, as evidenced by the upregulation of UPR-related elements in these innate immune cells upon exposure to supernatants from treated cancer cells [67]. In this system, induction of ER stress markers in myeloid cells correlated with their decreased ability to induce T cell responses, elevated expression of suppressive factors such as arginase I and prostaglandin E2 (PGE_2_), and upregulation of various cytokines including IL-6, IL-8, TNFα, and IL-23 [67] [10]. The impairment of myeloid cells exposed to supernatants from ER-stressed cancer cells to activate T cell responses was associated with a reduction in their antigen-presenting capacity [68]. Moreover, DCs conditioned in vitro with supernatants from ER-stressed cancer cells were transformed into MDSCs and facilitated tumor growth after adoptive transfer into tumor-bearing mice [10] (Fig. 1). While these studies suggested that ER-stressed cancer cells release soluble factors that more efficiently modulate immune cell function, it remained mechanistically and functionally elusive whether myeloid-intrinsic UPR factors were indeed responsible for the correlative changes described. Interestingly, administration of the ER stressor thapsigargin to tumor-bearing mice accelerated cancer progression and enhanced the accumulation and immunosuppressive capacity of MDSC, a process that could be attenuated upon in vivo treatment with the ER stress chemical chaperone, 4-PBA [9]. PERK has been implicated in blocking the effects of type 1 interferon potentially through direct regulation of the interferon receptor [69]. Previous results also showed that activation of PERK and the subsequent phosphorylation of eIF2α increased the activity of NF-kB by controlling the translation, but not the degradation, of the NF-kB inhibitor IkB [70]. Similarly, activation of IRE1α and ATF6 induced the phosphorylation of IkB and the subsequent activation of NF-kB in a manner dependent upon TRAF2 and Akt90, respectively [70, 71]. However, the potential interaction between the UPR and NF-kB in myeloid cells within tumors remains to be explored. These studies indicate that cancer cells undergoing ER stress can avidly modulate the phenotype of tumor-infiltrating myeloid cells.

### Cancer cell-intrinsic ER stress and immunogenic cell death (ICD)

**Fig. 2:**
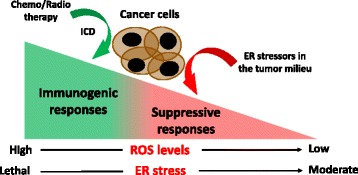
The severity of ER stress and the levels of ROS in cancer cells can determine the outcome of immune responses within the tumor milieu. Intense ER stress responses induced by chemo- or radiotherapy increase ROS in cancer cells to levels that can promote immunogenic cell death (ICD), thus enhancing anti-tumor immunity. Moderate but sustained ER stress responses in cancer cells support tolerogenic and immunosuppressive functions in tumor-infiltrating myeloid cells, a process that cripples anti-cancer immunity

Chemotherapeutic agents of the anthracycline family have been shown to trigger the UPR in cancer cells and this process was associated with the induction of immunogenic cell death (ICD), activation of myeloid cell function, and protective anti-tumor immunity [72]. Nonetheless, it remains unclear how the induction ER stress in malignant cells could result in the development of suppressive or immunogenic responses. ICD induction by ER stress appears to be mediated through a significant elevation of ROS levels and a subsequent activation of the NLRP3-inflammasome [4, 73]. However, the accumulation of ROS also remains as a major mechanism of T cell suppression by myeloid cells in tumors [74]. The fine balance between the levels of ROS and the specific ROS mediators could explain the opposite effects induced by stressed cancer cells on anti-tumor immunity (Fig. 2). Alternatively, the different consequences of tumor cells undergoing ER stress could also be explained by the simultaneous development of suppressive and immunogenic UPR in different subsets within the malignant cell population. Another plausible explanation is that moderate but sustained ER stress triggers immunosuppressive effects, whereas a robust/lethal UPR could result in ICD (Fig. 2). Interestingly, superior anti-tumor immune responses were observed in mice injected with BiP-deficient fibrosarcoma cells, presumably due to lethal overactivation of ER stress sensors that promotes ICD [75]. Hence, sustained ER stress responses occurring in transformed cells could promote immunosuppression, while the dramatic overactivation of the UPR upon acute chemo- or radiotherapy regimens may promote immunostimulatory responses (Fig. 2). Strikingly, however, XBP1 was recently shown to prevent ICD in metastatic colorectal cancer cells upon combination treatment with epidermal growth factor receptor blockers and chemotherapy [76].

### Intrinsic roles of the UPR in tumor-associated myeloid cells

Elevated expression of UPR mediators in tumors correlated with stage, aggressiveness, and low survival in patients with different malignancies. However, the link between the induction of ER stress in the tumor stroma and immunosuppression in individuals with cancer has not been appreciated over the last 10 years. Initial studies showed the role of UPR in the regulation of inflammation through modulation of the production of ROS and the activation of NF-kB, Jnk and IRF3 [1]. Most recently, however, various groups have demonstrated that sustained ER stress responses also act as crucial drivers of myeloid cell dysfunction in tumors [7, 8, 77].

**Fig. 3:**
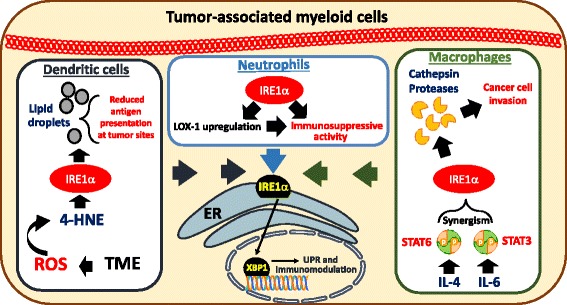
IRE1ɑ-XBP1 is one of the arms of UPR that polarizes tumor-infiltrating myeloid cells into highly immunosuppressive populations. Over activation of IRE1ɑ-XBP1 pathway by the byproduct adduct 4-hydroxy-trans-2-nonenal (4-HNE) in the tumor microenvironment (TME) shifts tumor-infiltrating dendritic cells towards a tolerogenic phenotype that promotes cancer cell growth. IRE1ɑ-XBP1 activation upregulates lectin-type oxidized LDL receptor-1 (LOX-1) that converts high density anti-tumor neutrophils to low density immunosuppressive polymorphonuclear myeloid cells (PMN-MDSCs). IL-4 and IL-6 signals synergize with IRE1ɑ-XBP1 to enhance the ability of tumor-associated macrophages to secret Cathepsin proteases, which facilitate cancer cell invasion and metastasis

IRE1α − XBP1 signaling is required for the optimal homeostatic differentiation of plasma cells, eosinophils and some DC populations [78–80]. Optimal TLR-driven pro-inflammatory cytokine production in macrophages has been demonstrated to be mediated by XBP1 [24]. In a model of acute lung injury, neutrophils infiltrating early lesions exhibited signs of ER stress, and XBP1 expression by this specific myeloid population was required for disease progression [81]. Interestingly, the potential role of this arm of the UPR in controlling the regulatory phenotype of tumor-associated myeloid cells has recently emerged as a key mediator of immune suppression in cancer (Fig. 3). In ovarian cancer, dysfunctional tumor-associated DCs (tDCs) showed robust expression of ER stress markers and sustained activation of the IRE1α − XBP1 arm of the UPR, compared with DCs residing in non-tumor locations [7]. Persistent ER stress responses in tDCs were provoked by intracellular ROS that promoted lipid peroxidation and subsequent generation of reactive aldehyde byproducts such as 4-hydroxynonenal (4-HNE), which modified several ER-resident chaperones and proteins [7]. Treatment of tDCs with ROS-scavenging vitamin E or hydrazine derivatives that avidly sequester 4-HNE ameliorated ER stress responses in tDC [7]. Conditional deletion of *Xbp1* in DCs resulted in delayed ovarian cancer progression and this process was mediated through the induction of protective T cell anti-tumor immunity. Additional experiments further confirmed that tDCs lacking XBP1 were immunostimulatory rather than tolerogenic. Mechanistically, abnormal activation of XBP1 metabolically reprogrammed DCs towards aberrant triglyceride biosynthesis and uncontrolled lipid accumulation, a process that was associated with reduced tDC antigen-presenting capacity. Interestingly, aberrant lipid accumulation and the production of oxidized fatty acids are common tolerogenic characteristics of tumor-infiltrating DCs and MDSCs [82–84]. Consistent with the immunogenic effects induced upon deleting or silencing *Xbp1* in tDCs, targeting lipid uptake or inhibiting key mediators of fatty acid oxidation has been shown to boost anti-cancer immunity by enhancing myeloid cell function in the tumor microenvironment [82–84]. While there is a clear interaction between the induction of ER stress and the metabolic reprogramming of myeloid cells in tumors, it remains unknown whether the tolerogenic effects induced by the accumulation of lipids in myeloid cells are solely mediated through IRE1α-XBP1 signaling or whether additional mediators participate in this process. Nevertheless, recent studies by Gabrilovich and colleagues have elegantly reinforced the crucial immunoregulatory role of aberrant IRE1α-XBP1 signaling in human cancer-associated myeloid cells [85]. In diverse human cancer specimens, upregulation of ER stress-related gene signatures and surface expression of the lectin-type oxidized LDL receptor-1 (LOX-1) distinguished high-density neutrophils from low-density immunosuppressive polymorphonuclear MDSCs (PMN-MDSCs). Strikingly, pharmacological induction of ER stress in human neutrophils rapidly triggered LOX-1 upregulation and transformed them into immunosuppressive cells in an IRE1α/XBP1-dependent manner. These recent studies indicate that the sustained activation of the IRE1α-XBP1 arm of the UPR promotes immunosuppression in cancer hosts by modulating the activity of tumor-associated DC, neutrophils, and MDSCs. Furthermore, a recent study showed that IRE1α-XBP1 signaling also shapes the pro-tumoral attributes of macrophages in cancer [86]. Through synergism between the IL-4 and IL-6 signaling pathways to activate IRE1α, tumor-associated macrophages acquire a secretory phenotype that enables the infiltration of metastatic cancer cells via Cathepsin proteases.

In addition to the role of IRE1α-XBP1 in the suppressive function of tumor-infiltrating myeloid cells, recent studies have demonstrated a major function for the UPR downstream target CHOP as a key regulator of MDSC activity and turnover in tumors [77]. While the pro-apoptotic effect CHOP has been appreciated for years, it is now evident that it can also regulate other cellular functions independent of the induction of apoptosis. In fact, CHOP expression controlled the polarization of macrophages into “alternatively activated” cells and directly regulated the expression of various cytokines, including IL-23, IL-1β, and IL-6 [87–89]. Interestingly, CHOP levels can be increased not only upon activation of the UPR, but also through other immunoregulatory mechanisms, including nutrient starvation, TLR agonists, and increased ROS accumulation, suggesting its common involvement in multiple stress pathways. Elevated CHOP expression was found in MDSCs infiltrating mouse and human tumors, which directly correlated with the ability of MDSC to impair T cell responses [8, 77]. Interestingly, the injection of CHOP-competent cancer cells into systemic *Chop*-deficient mice or *Chop*-null bone marrow chimeras resulted in a significant anti-tumor effect mediated by CD8^+^ T cells, suggesting the importance of hematopoietic-intrinsic CHOP in tumor growth and tumor-induced tolerance [77]. Furthermore, MDSCs isolated from tumor-bearing mice devoid of CHOP exhibited reduced ability to block T cell responses and impaired expression of major inhibitory pathways, while demonstrating an extraordinary ability to prime T cell proliferation and induce anti-tumor effects. Additional studies showed the potential role of CHOP in the regulation of C/EBPβ, a pathway known to globally regulate MDSC function. This resulted in an increased production of IL-6 that played a primary role in the effects induced by CHOP. Thus, the inhibition of CHOP could represent a major strategy to overcome the tolerogenic function of MDSCs and other myeloid suppressive cells in tumors. Similar to the effect of the endogenously produced ROS in the activation of IRE1α-XBP1 in tDCs [7], we found that pharmacological scavenging of ROS prevented the induction of CHOP in tumor-associated myeloid cells [77], suggesting the common role of ROS in the induction of UPR in cancer-infiltrating myeloid cells. Although the induction of CHOP after ER stress is primarily mediated through ATF4, it remains unknown the role of the CHOP-independent ATF4 effects in tumor-associated myeloid cells. Nevertheless, a seminal study by Condamine and colleagues showed the role of ER stress in the regulation of MDSC survival in tumors [8]. Induction of ER stress was detected preferentially in tumor-infiltrating MDSCs and promoted MDSC apoptosis through TNF-related apoptosis induced ligand receptor 2 (DR5) and caspase 8 activation [14]. Thus, DR5 agonists could be considered as potential strategies for controlling MDSC generation in cancer. Interestingly, deletion of *Chop* also regulated MDSC turnover, as a delayed MDSC apoptosis and extended MDSC survival rates were found in tumor-infiltrating MDSCs lacking this UPR mediator, compared with CHOP-sufficient controls [77]. Taken together, these recent findings suggest that ER stress responses driven by IRE1α-XBP1 and CHOP play a major role in the regulation of myeloid cell activity and survival in tumors. It remains elusive, however, whether the ATF6 arm of the UPR also contributes to myeloid cell dysfunction in cancer.

### Therapeutic approaches to overcome detrimental ER stress responses in tumor-associated myeloid cells

Since the UPR appears to regulate anti-tumor immunity while promoting the intrinsic aggressiveness of malignant cells, it is conceivable that therapies aimed at attenuating ER stress or targeting UPR mediators may have a potent double-whammy effect against cancer. Chemical chaperones that prevent ER stress, such as TUDCA and 4-PBA, have shown promising therapeutic effects in preclinical cancer models. However, the consequence of treatment with these compounds on the global tumor immunoenvironment remains unknown. Additional efforts have been made to develop specific small-molecule inhibitors or nanoparticle-encapsulated siRNAs targeting UPR mediators. Compounds inhibiting the endoribonuclease domain of IRE1α, including STF-083010, 3-ethoxy-5,6-dibromosalicylaldehyde, 4μ8C, MKC-3946, toyocamycin, and B-I09, can block *Xbp1* splicing and activation in a dose dependent manner, especially in vitro [90]. Some of these compounds have been tested in vivo and demonstrated anti-tumor effects by directly affecting the cancer cell. While the immunotherapeutic capacity of these inhibitors has not been tested in vivo, delivery of nanoparticles encapsulating *Xbp1*-targeting siRNA into mice bearing metastatic ovarian carcinoma transformed tDCs into highly immunogenic cells capable of inducing protective T cell responses that extend host survival [7]. Treatment of tumor-bearing mice with the PERK small-molecule inhibitors GSK2656157 or GSK2606414 has also resulted in significant anti-tumor effects [49, 91], but it remains unknown whether these compounds could additionally relieve immunosuppression in the tumor microenvironment by controlling activation in myeloid cells, without inducing systemic toxicity. While these studies suggest the potential of targeting the UPR in cancer, disruptive medicinal chemistry approaches are urgently needed to generate more selective, potent and stable inhibitors of ER stress sensors for in vivo use.

A major impediment to the success of current immunotherapies is the accumulation of suppressive myeloid cells that prevent the generation and expansion of tumoricidal T cells [92]. Therapies based on targeting UPR mediators could be potentially used to reprogram suppressive myeloid populations into cells that activate anti-tumor immunity in situ. These approaches could be useful to relieve or diminish tumor-induced immunosuppression prior to treatment with other immunotherapies such as checkpoint blockade, adoptive T cell transfer or therapeutic vaccination. In summary, future studies on the role of the UPR in tumor-associated myeloid cells are expected to have a significant impact in the development of new immunotherapies that more effectively confront lethal cancers in the clinic.

## Conclusions

Controlling the accumulation and detrimental activity of immunosuppressive myeloid cells in cancer patients emerges as a fundamental requirement for the success of cancer immunotherapies. However, interventions that effectively and permanently abolish the major regulatory effect or the accumulation of myeloid cells in tumors are lacking. Sustained ER stress responses have been demonstrated to promote malignant progression and metastasis. Further, recent studies revealed an additional role for endogenous ER stress and the UPR in regulating the function, expansion and differentiation of suppressive myeloid cells in cancer hosts. The activation of the UPR in myeloid cells can directly occur in response to the stressful tumor microenvironment or may be transmitted from neighboring ER-stressed cancer cells. Since disabling some ER stress sensors and UPR mediators can reprogram suppressive myeloid cells into cells that induce protective anti-tumor immunity, new interventions capable of controlling this pathway in vivo could improve the effectiveness of emerging cancer immunotherapies. We therefore propose that understanding the cellular and molecular effects of ER stress in tumor-associated myeloid cells will be crucial for developing more rational and hopefully definitive immunotherapies against lethal cancers.

## Abbreviations

4-HNE: 4-hydroxynonenal
4-PBA: 4-Phenylbutyric acid
ASK1: Apoptosis-signal-regulating kinase
ATF4: Activating transcription factor 4
ATF6: Activating transcription factor 6
C/EBPβ: CAAT/enhancer binding protein beta
CHOP: C/EBP homologous protein
CML: Chronic myeloid leukemia
DAG: Diacyglycerol
DCs: Dendritic Cells
DR5: TNF-related apoptosis induced ligand receptor 2
eIF2α: Eukaryotic translation initiation factor 2 alpha
ER: Endoplasmic reticulum
FOXO: Forkhead box O protein
GCN2: Nutrient starvation activated kinase
HRI: Hemin-regulated inhibitor
ICD: Immunogenic cell death
IRE1: Inositol-requiring enzyme 1
JNK: c-Jun N-terminal kinase
LOX-1: Lectin-type oxidized LDL receptor-1
MDSCs: Myeloid-derived suppressor cells
Nrf2: NF-E2-related factor 2
PERK: Pancreatic ER kinase (PKR)-like ER kinase
PGE_2_: Prostaglandin E2
PKR: Double-stranded RNA-dependent protein kinase
PMN-MDSCs: Polymorphonuclear MDSCs
RIDD: Regulated IRE1α-dependent decay
ROS: Reactive oxygen species
tDCs: Tumor-associated DCs
TRAF2: TNF-receptor-associated factor 2
TUDCA: Tauroursodeoxycholic acid
UPR: Unfolded Protein Response
XBP1: X-box binding protein 1
